# Microbial Composition and Co-occurrence Patterns in the Gut Microbial Community of Normal and Obese Mice in Response to Astaxanthin

**DOI:** 10.3389/fmicb.2021.671271

**Published:** 2021-09-06

**Authors:** Yuan Gao, Fang Liu, Robert W. Li, Chunjun Li, Changhu Xue, Qingjuan Tang

**Affiliations:** ^1^Laboratory of Food Science and Human Health, College of Food Science and Engineering, Ocean University of China, Qingdao, China; ^2^Laboratory of Animal Genomics and Improvement, United States Department of Agriculture, Agriculture Research Service (USDA-ARS), Beltsville, MD, United States; ^3^Laboratory of Marine Drugs and Biological Products, Pilot National Laboratory for Marine Science and Technology, Qingdao, China

**Keywords:** astaxanthin, microbial composition, global network, modularity, co-occurrence patterns

## Abstract

The changes and interaction of gut microbiota, which respond to dietary supplements, play critical roles on improving human health. The modulating effect of astaxanthin on gut microbiota has been reported. However, little is known about the co-occurrence patterns among microbial taxa in response to astaxanthin. In this study, the gut microbial composition, co-occurrence patterns, and microbial correlations with physiological parameters in astaxanthin-fed normal and obese mice were studied. Astaxanthin altered the microbial composition and co-occurrence patterns in normal and obese mice. Furthermore, astaxanthin gave more profound impacts on microbiota in obesity when compared with normal mice. In group A (normal or obese mice supplemented with astaxanthin), the abundance of *Acinetobacter* was decreased, and *Alistipes* was increased by astaxanthin, which also occurred in the MA group (obese mice supplemented with astaxanthin). An operational taxonomic unit (OTU) (GreenGeneID# 4029632) assigned to the genus *Bacteroides* acted as a connector in the global network of A and MA groups. It may play critical roles in bridging intimate interactions between the host and other bacteria intervened by astaxanthin. Several modules correlated with physiological parameters were detected. For example, modules A12 and MA10 were significantly and negatively correlated with lipopolysaccharide (LPS) and fasting blood glucose (FBG) levels, respectively. A positive correlation was found between the node connectivity of the OTUs belonging to Clostridiaceae with LPS in obese mice, which indicated the role of Clostridiales as a potential pathological marker. Our findings provided a new interpretation of the role of astaxanthin in health and may contribute to further research on microbial community engineering.

## Introduction

Astaxanthin is a kind of xanthophyll carotenoid, which can be widely found in marine organisms, such as algae, fish, shrimps, crabs, and other organisms (Davinelli et al., 2018). Due to the strong antioxidant capacity and great improvement effect on metabolic syndromes, astaxanthin has been favored as a health food supplement by obese consumers, and various studies have confirmed this. It has been reported that the addition of astaxanthin reduced hepatic lipid accumulations in high-fat-fed mice, of which the pathway of peroxisome proliferator-activated receptor (PPAR) alpha was activated and the pathway of PPAR gamma was inhibited (Jia et al., 2016). Kim et al. (2017) found that astaxanthin inhibited inflammation and fibrosis in the liver and adipose tissue in obese mice, and the mitochondrial fatty acid oxidation function was enhanced in the skeletal muscle. In addition, astaxanthin has been found to play roles in improving insulin resistance and hepatic inflammation *via* increasing M2-dominant macrophages/Kupffer cells and reducing CD4(+) and CD8(+) T cell recruitment in the liver (Ni et al., 2015).

In addition to the mechanisms mentioned above, the gut microbiota may play a role in the ameliorating effects of astaxanthin under obese condition. The modulating effect of astaxanthin on gut microbiota has been reported in various studies. Compared to mice with alcoholic fatty liver disease, the supplement of astaxanthin significantly altered the composition of gut microbiota at different levels—for example, the abundance of microbiota from the phylum Proteobacteria and the microbiota, such as *Butyricimonas*, *Bilophila*, and *Parabacteroides*, from genus level was decreased (Liu et al., 2018). In another study, astaxanthin was found to have a regulating effect on immunoglobulin A in mice, and the mechanism may be related to the modulating effects of astaxanthin on gut microbiota, especially on Bifidobacterium (Lyu et al., 2018). In addition, the bioaccessibility of astaxanthin was lower than 50% in normal body and even lower in obese body (<10%), which increased the possibility of the interaction of astaxanthin and gut microbiota (Gao et al., 2020b).

Although the modulating effects of astaxanthin on gut microbiota in obese bodies have been reported previously, little is known about the co-occurrence patterns of different taxa of gut microbiota in response to astaxanthin. The interactions among the microbiota play a great role in determining the structure and function of the microbial communities. Exploring the co-occurrence feature of microbiomes from a network perspective is very important for understanding and revealing the role that the gut microbiota plays after dietary supplement intervention (Ma et al., 2020). In addition, the correlation between the microbial modules and the representative physiological indicators, such as fasting blood glucose (FBG) and lipopolysaccharide (LPS), is of great significance for exploring the effects of astaxanthin on the body. In this study, after the supplement of astaxanthin in normal and obese mice, the interactions among different microbial taxa were studied, and the keystone microbial species that may be correlated with the significantly changed physiological indicators, such as FBG and LPS, were studied using co-occurrence network tools.

## Materials and Methods

### Materials

*Haematococcus pluvialis* extract (Yunnan Alphy Biotech. Co., Ltd., Chuxiong, Yunnan Province, China) was used as the source of astaxanthin in this study. The concentration of astaxanthin in the *H. pluvialis* extract was 10% (w/w) as detected by high-performance liquid chromatography with diode array detection. The total astaxanthin content was composed of 71% astaxanthin monoesters, 28% astaxanthin diesters, and <1% free-form astaxanthin as reported previously (Gao et al., 2020b).

### Animal Experiment

The animal experiment was previously described (Gao et al., 2020b). Briefly, after an adaption period of 1 week, C57BL/6J mice (male, 6 weeks old, 20–22 g) were randomly divided into two groups fed with normal control diet and high-fat diet (HFD), respectively, for 8 weeks to establish normal and obese mouse models. The diets were prepared according to the feed formula provided by Research Diets (New Brunswick, NJ, United States; Supplementary Table 1). After the models were established, the mice were randomly divided into four groups: normal mice + corn oil (group NO, *n* = 7), normal mice supplemented with astaxanthin (60 mg/kg body weight) with corn oil as vehicle for 30 consecutive days (group NA, *n* = 9), obese mice + corn oil (group MO, *n* = 8), and obese mice supplemented with astaxanthin (60 mg/kg body weight) with corn oil as vehicle for 30 consecutive days (group MA, *n* = 10). After overnight fasting, the mice were sacrificed, and blood samples and colon contents were collected. The serum glucose level and serum LPS level were determined by commercial kits. This study was conducted in strict accordance with the Guide for the Care and Use of Laboratory Animals of the National Institutes of Health. All experiment procedures were approved by the Animal Ethics Committee of Ocean University of China (approved protocol no: SPXY2017050402).

### 16S rRNA Sequencing and Data Analysis

The microbial genomic DNA from the colon contents of the NO, NA, MO, and MA groups was extracted with a QIAamp DNA stool Mini kit (Qiagen, cat. no. 51604) (Gao et al., 2020a). Gel electrophoresis was used to verify the integrity of the total DNA, and the concentration of DNA was quantified by Nanodrop 2000 (Thermo Fisher Scientific, United States). The DNA samples that did not meet the required quality or concentration were deleted. 16S rRNA sequencing and analysis were used for obtaining the gut microbiota profile. The V3–V4 regions of the 16S rRNA gene were selected for amplification as previously reported (Li et al., 2016). The sequencing was done on 7 samples from the NO group, 9 samples from the NA group, 8 samples from the MO group, and 10 samples from the MA group, respectively. The total DNA was amplified with specific primers (forward primer, 341/357F: NNNNCCTACGGGNGGCWGCAG; reverse primer, 805/785R: GACTACHVGGGTATCTAATCC) tagged with barcodes. The PCR reactions were carried out with Phusion high-fidelity PCR Master Mix (New England Biolabs, Ipswich, MA, United States). The concentration of the library pool was quantified by a BioAnalyzer DNA chip kit (Agilent), and the library was sequenced by an Illumina sequencer (Marques et al., 2017).

The assignment of taxonomy was based on the GreenGene database. The significantly changed microbial taxa and Kyoto Encyclopedia of Genes and Genomes (KEGG) pathways after the dietary intervention were identified by linear discriminant analysis (LDA) effect size (LEfSe) algorithm (Segata et al., 2011). Furthermore, the Phylogenetic Investigation of Communities by Reconstruction of Unobserved States (PICRUSt) algorithm (Langille et al., 2013) was used with default parameters to predict gene contents and metagenomic functional information.

### Network Construction and Visualization

A random matrix theory-based pipeline was used to construct the global networks (Zhou et al., 2011; Deng et al., 2012).^1^ Considering the huge impact of operational taxonomic unit (OTU) sparsity on the accuracy of network inference (Weiss et al., 2016), the OTUs detected in <50% of samples were excluded. In this study, the threshold values were 0.76 for group O (mice without astaxanthin, i.e., NO and MO groups; *n* = 15), 0.70 for A (mice supplemented with astaxanthin, i.e., NA and MA groups; *n* = 19), 0.92 for NO (*n* = 7), 0.87 for NA (*n* = 9), 0.92 for MO (*n* = 8), and 0.85 for MA (*n* = 10), respectively. The module separation was implemented by the fast-greedy modularity optimization procedure. A scatter plot used for displaying the topological roles of the OTU nodes in the co-occurrence was depicted by the within-module degree (Zi) and among-module connectivity (Pi) values. The module–eigengene analysis was performed by using Pearson correlation analysis on the greedy modularity optimization dataset and environmental traits (FBG and LPS levels in this analysis); small modules with less than five members were ignored. The OTU significances (GS) with environmental traits was calculated, and Mantel test was then used to check the correlations between GS and network connectivity, as described (Zhou et al., 2011). The software Cytoscape v3.6.1 was used for the visualization of the networks (Shannon et al., 2003).

### Statistical Analysis

The results are presented as mean ± standard error of the mean (SEM). The analysis of statistical significance was conducted by one-way analysis of variance (ANOVA) using PASW Statistics 18 software. A *p*-value < 0.05 was considered statistically significant, and a *p*-value < 0.01 was considered extremely significant.

## Results

### Astaxanthin Modulated Gut Microbiota Under an HFD

**FIGURE 1:**
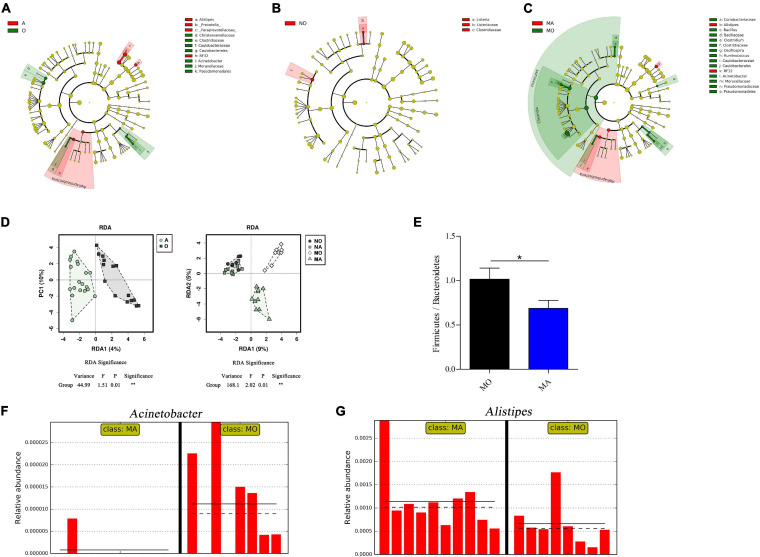
Selected microbial taxa with significant different abundance in samples using linear discriminant analysis (LDA) effect size analysis. Taxa meeting a significant LDA threshold value >2.0 and its corresponding taxonomic cladogram are depicted in **(A)** A vs. O groups (red: A-enriched taxa; green: O-enriched taxa), **(B)** NA vs. NO groups (red: NO-enriched taxa), and **(C)** MA vs. MO groups (red: MA-enriched taxa; green: MO-enriched taxa). **(D)** Redundancy analysis between A and O (left) and among NO, NA, MO, and MA groups (right). **(E)** The ratio of Firmicutes to Bacteroides. **(F)** *Acinetobacter* and **(G)** *Alistipes* are selected microbial genera displaying significant differences in their relative abundance in the gut microbiota between MA and MO. *x*-axis, individual samples; *y*-axis, relative abundance; straight line, group mean abundance; dotted line, median; O, normal or obese mice + corn oil vehicle; A, normal or obese mice + astaxanthin; NO, normal mice + corn oil vehicle; NA, normal mice + astaxanthin; MO, obese mice + corn oil vehicle; MA, obese mice + astaxanthin. Astaxanthin was dissolved in corn oil at a daily dose of 60 mg/kg body weight (astaxanthin equivalents) and supplemented for 30 days. *p < 0.05.

To assess the effects of astaxanthin on gut microbiota under normal and obese conditions, 16S rRNA gene sequencing of the V3–V4 regions was performed with the colon content of the four groups of mice. An average of 140,447 ± 55,110 reads per sample was generated from the colon content. There were 2,423 OTUs identified from the sequencing data (Supplementary Table 2). In order to make the analysis of the effects of astaxanthin on the gut microbiota more meaningful, we divided the samples into two groups, namely, the mice administered with astaxanthin (group A, i.e., NA and MA groups) and the mice without astaxanthin (group O, i.e., NO and MO groups), of which the sample range was increased. LEfSe analysis detected 14 taxa that were significantly impacted in group A compared to group O, and four and 21 taxa were significantly impacted by astaxanthin under healthy and obese conditions (absolute log_10_ LDA score > 2.0), respectively (Figures 1A–C). The changes in the microbiota induced by astaxanthin became more profound under the obese condition. The redundancy analysis (Figure 1D) revealed significant differences in the composition of gut microbiota between group A and group O and the MA and MO groups, while no significance was observed between the NA and NO groups. At the phylum level, the gavage of astaxanthin significantly decreased the abundance of Firmicutes in the MA group compared to MO (log_10_ LDA score = 4.7), and a significantly lower Firmicutes/Bacteroidetes ratio was observed in the MA group (Figure 1E). At the genus level, at least three named genera were significantly impacted by astaxanthin in group A compared to group O. Among them, the abundance of *Acinetobacter* was decreased, and the abundance of *Alistipes* was increased by astaxanthin, which also occurred in the MA group compared to MO (Figures 1F,G). Only one genus (*Listeria*) was observed to have been significantly impacted by astaxanthin in the NA group compared to NO.

The abundance of 28 and 42 OTUs was affected by astaxanthin under healthy and obese conditions, respectively. An obvious change was observed in the gut microbiota with HFD. The abundance of at least 139 OTUs was significantly altered in the MO group. Specifically, 17 OTUs assigned to the order Clostridiales were significantly increased in the MO group compared to NO, while after the supplement of astaxanthin, they were significantly decreased in the MA group. Astaxanthin increased the abundance of 15 OTUs and decreased the abundance of 40 OTUs in group A compared to group O. Intriguingly, some OTUs displayed unidirectional changes in their abundance under different body conditions (Table 1)—for example, OTU #780650, assigned to Clostridiaceae, which was significantly decreased in group A compared to group O, was also significantly decreased by astaxanthin under healthy and obese conditions, respectively. An OTU belonging to the family Ruminococcaceae (GreenGene ID #349459), which was significantly increased by astaxanthin in group A, was also significantly increased under healthy condition. An OTU (GreenGeneID #175458) belonging to the family S24-7, an OTU (GreenGeneID #325850) from the order RF32, and an OTU (GreenGeneID #4331760) assigned to *Alistipes indistinctus*, which were likewise significantly increased in their abundance by astaxanthin in group A, were also significantly increased under obese condition.

**TABLE 1:** Operational taxonomic units (OTUs) significantly impacted by astaxanthin in the gut microbiome in mice.

OTU_ID (GreenGene)	Annotation	O	A	NO	NA	MO	MA	Significant
322062	(Lachnospiraceae)	0.0483 ± 0.0094	0.0628 ± 0.0104	0.0170 ± 0.0057	0.0469 ± 0.0102	0.0756 ± 0.0090	0.0771 ± 0.0167	b
589071	*Bacteroides uniformis*	0.1475 ± 0.0525	0.0666 ± 0.0401	0.0757 ± 0.0662	0.1315 ± 0.0813	0.2104 ± 0.0765	0.0081 ± 0.0057	c
780650	(Clostridiaceae)	1.1960 ± 0.3487	0.0331 ± 0.0156	0.4168 ± 0.2035	0.0008 ± 0.0003	1.8790 ± 0.5314	0.0621 ± 0.0269	abc
349459	(Ruminococcaceae)	0.4052 ± 0.0986	0.8961 ± 0.1818	0.3545 ± 0.1466	1.0500 ± 0.2349	0.4496 ± 0.1404	0.7577 ± 0.2775	ab
175458	(S24-7)	0.0002 ± 0.0000	0.3072 ± 0.0852	0.0002 ± 0.0001	0.2733 ± 0.1347	0.0002 ± 0.0001	0.3377 ± 0.1132	ac
325850	(RF32)	0.0049 ± 0.0028	0.0706 ± 0.0306	0.0093 ± 0.0057	0.0424 ± 0.0155	0.0010 ± 0.0010	0.0960 ± 0.0566	ac
4331760	*Alistipes indistinctus*	0.0676 ± 0.0099	0.1065 ± 0.0144	0.0728 ± 0.0081	0.1042 ± 0.0204	0.0631 ± 0.0176	0.1085 ± 0.0212	ac

*O, normal or obese mice + corn oil vehicle; A, normal or obese mice + astaxanthin; NO, normal mice + corn oil vehicle; NA, normal mice + astaxanthin; MO, obese mice + corn oil vehicle; MA, obese mice + astaxanthin. Astaxanthin was dissolved in corn oil at a daily dose of 60 mg/kg body weight (astaxanthin equivalents) and supplemented for 30 days. The numbers denote the relative abundance. Data are presented as mean ± SEM. Significant means that the abundance of the OTU was changed at a cutoff value of the absolute log_10_ linear discriminant analysis scores >2.0 between the two contrast groups using linear discriminant analysis effect size algorithm (a = O vs. A; b = NO vs. NA; c = MO vs. MA).*

### Microbial Pathways Impacted by Astaxanthin

**FIGURE 2:**
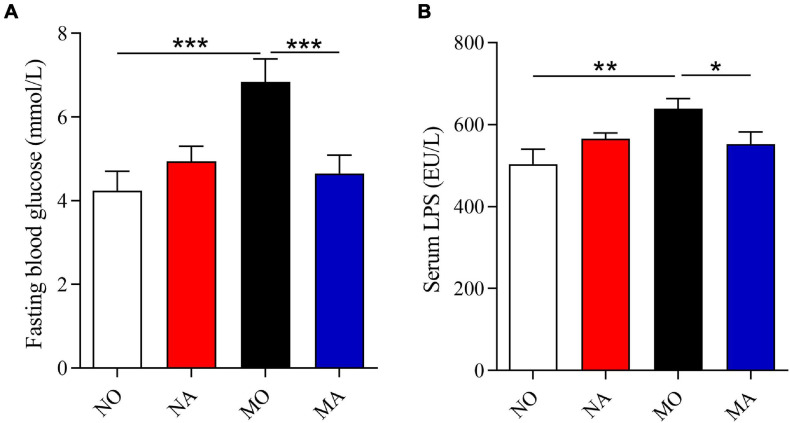
Astaxanthin ameliorated the glucose level and circulating inflammation under high-fat and high-sucrose diet. **(A)** Fasting blood glucose level. **(B)** Serum lipopolysaccharide level. NO, normal mice + corn oil vehicle; NA, normal mice + astaxanthin; MO, obese mice + corn oil vehicle; MA, obese mice + astaxanthin. Astaxanthin was dissolved in corn oil at a daily dose of 60 mg/kg body weight (astaxanthin equivalents) and supplemented for 30 days. The values are presented as mean ± SEM. *p < 0.05; **p < 0.01; ***p < 0.001.

Using the PICRUSt (Langille et al., 2013), the functional potential of astaxanthin on normal and obese bodies was analyzed from the 16S rRNA gene sequence data. A total of 328 KEGG pathways were predicted. No significant effect of astaxanthin on the KEGG pathways was observed in group A compared to group O, but in obese bodies, astaxanthin was found to have effects on a range of biological functions when compared with MO (Table 2)—for example, astaxanthin significantly affected certain pathways related to carbohydrate metabolism, such as polyketide sugar unit biosynthesis (log_10_ LDA score = 2.20), butanoate metabolism (log_10_ LDA score = 2.22), and propanoate metabolism (log_10_ LDA score = 2.09). The abundance of genes involved in membrane and intracellular structural molecules, LPS biosynthesis, and LPS biosynthesis proteins, which were significantly decreased in the MO group compared to NO, was significantly increased by astaxanthin in the MA group. The FBG level and circulating LPS level were significantly affected by astaxanthin in the MA group, while no effects were observed under normal conditions (Figure 2), which was consistent with the prediction results of the effects of astaxanthin on the biological function in the fecal microbial community.

**TABLE 2:** Selected biological pathways and functional categories of gut microbiota displaying differences in relative abundance of gene function (%).

Pathway	NO	NA	MO	MA	Significant
Polyketide sugar unit biosynthesis	0.2549 ± 0.0081	0.2557 ± 0.0064	0.2219 ± 0.0074	0.2423 ± 0.0077	ab
Membrane and intracellular structural molecules	0.6767 ± 0.0231	0.6767 ± 0.0252	0.5578 ± 0.0241	0.6261 ± 0.0200	ab
Sulfur metabolism	0.2609 ± 0.0110	0.2600 ± 0.0073	0.2342 ± 0.0038	0.2477 ± 0.0038	ab
Lipopolysaccharide biosynthesis proteins	0.5107 ± 0.0227	0.5187 ± 0.0220	0.4152 ± 0.0196	0.4797 ± 0.0214	ab
Lipopolysaccharide biosynthesis	0.3948 ± 0.0227	0.4016 ± 0.0217	0.3100 ± 0.0186	0.3689 ± 0.0195	ab
Butanoate metabolism	0.6556 ± 0.0096	0.6649 ± 0.0101	0.6899 ± 0.0094	0.6592 ± 0.0078	ab
Propanoate metabolism	0.4513 ± 0.0076	0.4525 ± 0.0047	0.4740 ± 0.0052	0.4511 ± 0.0054	ab

*NO, normal mice + corn oil vehicle; NA, normal mice + astaxanthin; MO, obese mice + corn oil vehicle; MA, obese mice + astaxanthin. Astaxanthin was dissolved in corn oil at a daily dose of 60 mg/kg body weight (astaxanthin equivalents) and supplemented for 30 days. The values are presented as mean ± SEM. Significant means that the abundance of the genes involved in the pathways were changed at a cutoff value of the absolute log_10_ linear discriminant analysis scores >2.0 between the two contrast groups using linear discriminant analysis effect size algorithm (a = NO vs. MO; b = MO vs. MA).*

### Visualization of the Topological Roles of Individual Nodes and Their Possible Ecological Roles

There were 1,995 (group O), 2,003 (group A), 1,413 (group NO), 1,545 (group NA), 1,693 (group MO), and 1,550 (group MA) OTUs used for the global network construction for the six groups, respectively. The topological properties of the global networks of the six groups are described in Supplementary Table 3. The number of nodes (links) in the microbial co-occurrence networks was 381 (581) in group O, 325 (411) in group A, 447 (751) in NO, 420 (493) in NA, 566 (866) in MO, and 393 (587) in MA, respectively. The gavage of astaxanthin altered the composition and structure of the network. The supplement of astaxanthin altered 190, 161, and 302 OTUs in the global network of group O, group NO, and group MO, which was equivalent to 50, 36, and 53% of all nodes, respectively.

**FIGURE 3:**
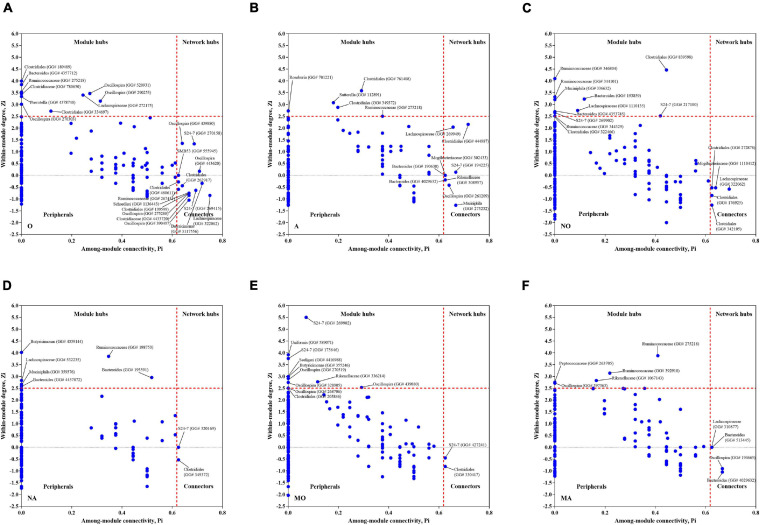
Scatter plot showing the distribution of operational taxonomic units (OTUs) based on their topological roles in the network. **(A)** Group O. **(B)** Group A. **(C)** Group NO. **(D)** Group NA. **(E)** Group MO. **(F)** Group MA. The detailed information for each OTU can be found in Supplementary Table 2. Each dot represents an OTU. Zi, within-module connectivity; Pi, among-module connectivity; O, normal or obese mice + corn oil vehicle; A, normal or obese mice + astaxanthin; NO, normal mice + corn oil vehicle; NA, normal mice + astaxanthin; MO, obese mice + corn oil vehicle; MA, obese mice + astaxanthin. Astaxanthin was dissolved in corn oil at a daily dose of 60 mg/kg body weight (astaxanthin equivalents) and supplemented for 30 days.

Different nodes play distinct topological roles in the network (Guimerà et al., 2007). The topological roles of the OTUs identified in these networks are shown in Figure 3. The majority of the nodes (96.6%) from the six global networks were peripherals (Zi ≤ 2.5 and Pi ≤ 0.62), whose links were mostly inside their modules. Among the peripherals, between 77.0 and 90.3% were with a Pi value of 0, which means that they had no links with the outside modules. Between 5 and 11 nodes (1.3–2.6%) were module hubs in the networks, which have a high *Z* (Zi > 2.5) but a low *P*-value (Pi ≤ 0.62), and highly connected to other nodes in their own modules—for example, an OTU (GreenGeneID# 589071) belonging to the species *uniformis* from the genus *Bacteroides* and two OTUs (GreenGeneID# 269902 and 175646, respectively) from the family S24-7 were identified as module hubs in group MO. Among them, the abundance of OTU# 589071 was significantly decreased by astaxanthin in the MA group (Table 1). The module hubs in each global network were distinctly different, and there were only two nodes which existed as module hubs in different groups. An OTU (GreenGeneID# 269902) assigned to the family S24-7 was observed to serve as a module hub in both the NO and MO groups. Of note is that an OTU (GreenGeneID# 275218) from Ruminococcaceae behaved like module hubs in both the MA and A groups. A few nodes acting as a connector, which have a low *Z* but a high *P*-value and highly linked to several modules, were shown in *Z*–*P* plots—for example, an OTU (GreenGeneID# 322062) from the family Lachnospiraceae was identified as a connector species in group NO, which was significantly increased by astaxanthin in NA group (LDA score > 2.0). An OTU (GreenGeneID# 4029632) belonging to the genus *Bacteroides* existed as a connector in both the MA and A groups. However, there were no network hubs observed from the analysis of the networks.

### The Correlations Between Modules and Physiological Parameters

**FIGURE 4:**
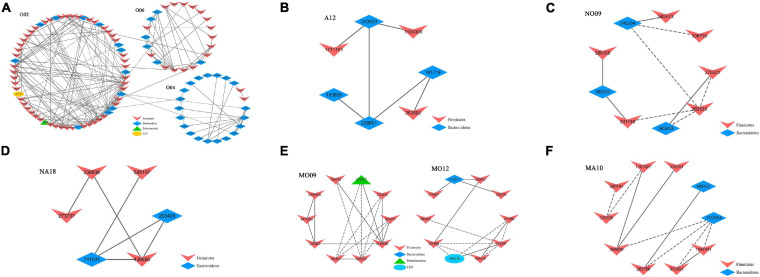
Select modules displaying a strong correlation with fasting blood glucose (FBG) or/and lipopolysaccharide (LPS) level. **(A)** The modules identified from group O displayed a significant correlation with either FBG (module O06) or LPS (module O04) alone or both (module O02). **(B)** The module A12 identified from group A showed a significant negative correlation with LPS. **(C)** The module NO09 identified from group NO displayed a strong negative correlation with LPS. **(D)** The module NA18 identified from group NA displayed a strong positive correlation with FBG. **(E)** The modules MO12 and MO09 identified from group MO showed a positive correlation with FBG (MO12) and LPS (MO09), respectively. **(F)** The module MA10 identified from group MA displayed a negative correlation with FBG. The interactions among different nodes (operational taxonomic units, OTUs) within a module are shown. Solid line, positive correlation; dashed line, negative correlation. The color of each node (OTU) indicated the phylum that this OTU was assigned to. The detailed annotation of each OTU node can be found in Supplementary Table 2. O, normal or obese mice + corn oil vehicle; A, normal or obese mice + astaxanthin; NO, normal mice + corn oil vehicle; NA, normal mice + astaxanthin; MO, obese mice + corn oil vehicle; MA, obese mice + astaxanthin. Astaxanthin was dissolved in corn oil at a daily dose of 60 mg/kg body weight (astaxanthin equivalents) and supplemented for 30 days.

The module-based microbiota were studied to correlate the physiological parameters (FBG and LPS) with the individual modules (Table 3). In group O, at least three modules (Figure 4A) were strongly correlated with the two physiological traits. Among them, module O06 showed a significant positive correlation with the FBG level (*r* = 0.56; *p* = 0.0300), while module O04 exhibited a significantly negative correlation with LPS (*r* = −0.80; *p* = 0.0003). Module O02 was significantly positively correlated with both FBG and LPS values (*p* = 0.02 and 0.01, respectively). Module O02 included 62 OTUs, most of which were from Firmicutes, and 47 members were belonging to Clostridiaceae. After the treatment of astaxanthin (group A), module A12 (Figure 4B) was observed to be negatively correlated with LPS (*r* = −0.69; *p* = 0.0010). There were seven members contained in module A12, four of which were from the phylum Bacteroidetes, and three were from Firmicutes. All four members of the phylum Bacteroidetes were belonging to the order Bacteroidales, and all three members of the phylum Firmicutes were from the order Clostridiales. In the normal group (NO), module NO09 (Figure 4C) was observed to have a significant negative correlation with LPS value (*r* = −0.91; *p* = 0.0050). In group NA, module NA18 (Figure 4D) was strongly positively correlated with FBG (*r* = 0.82; *p* = 0.0060). Under obese condition, modules MO12 and MO09 (Figure 4E) showed a significant positive correlation with FBG (*r* = 0.78; *p* = 0.0200) and LPS (*r* = 0.72; *p* = 0.0500), respectively. Module MO09 contained a total of 10 members, of which at least 3 OTUs assigned to Clostridiales were significantly increased in obese bodies compared to the NO group. After the gavage with astaxanthin in obese bodies, module MA10 (Figure 4F) was negatively correlated with FBG value (*r* = −0.71; *p* = 0.0200). Module MA10 included 10 OTUs, 3 of which were from the family Lactobacillaceae, and three were assigned to Ruminococcaceae.

**TABLE 3:** The correlations between the eigengene values of select modules and physiological traits.

Module	Physiological parameters	*r*	*P*-value	Module members
Global network/group: O				
02	FBG	0.58	0.0200	62
06	FBG	0.56	0.0300	22
02	LPS	0.64	0.0100	62
04	LPS	–0.80	0.0003	22
Global network/group: A				
12	LPS	–0.69	0.0010	7
Global network/group: NO				
09	LPS	–0.91	0.0050	9
Global network/group: NA				
18	FBG	0.82	0.0060	6
Global network/group: MO				
12	FBG	0.78	0.0200	10
09	LPS	0.72	0.0500	10
Global network/group: MA				
10	FBG	–0.71	0.0200	10

*O, normal or obese mice + corn oil vehicle; A, normal or obese mice + astaxanthin; NO, normal mice + corn oil vehicle; NA, normal mice + astaxanthin; MO, obese mice + corn oil vehicle; MA, obese mice + astaxanthin; FBG, fasting blood glucose; LPS, lipopolysaccharide. Astaxanthin was dissolved in corn oil at a daily dose of 60 mg/kg body weight (astaxanthin equivalents) and supplemented for 30 days.*

The selected correlationships between the node connectivity of some taxa and physiological parameters in the six groups (O, A, NO, NA, MO, and MA) are listed in Table 4. After the treatment of astaxanthin in group A, a positive correlation was found between the node connectivity of the phylum Proteobacteria and the LPS value. In group NO, the node connectivity of the OTUs assigned to the family Rikenellaceae showed a positive correlation with both FBG and LPS values. In group MO, the family Clostridiaceae exhibited a positive correlation with the LPS value. After the treatment of astaxanthin in obese bodies, a positive correlation was observed between the node connectivity of the LPS value and the OTUs belonging to the genus *Dorea*.

**TABLE 4:** The partial Mantel test revealed the correlation between the node connectivity of some taxa and the operational taxonomic unit significance of physiological traits in microbial co-occurrence networks.

Treatment group	Physiological parameter	Taxon (level)	*r* (correlation coefficient)	Significance (probability)
A	LPS	Proteobacteria (phylum)	0.8929	0.0833
NO	FBG	Rikenellaceae (family)	0.7246	0.0170
NO	LPS	Rikenellaceae (family)	0.6053	0.0420
MO	LPS	Clostridiaceae (family)	0.6565	0.0020
MA	LPS	*Dorea* (genus)	0.9436	0.0833

*A, normal or obese mice + astaxanthin; NO, normal mice + corn oil vehicle; MO, obese mice + corn oil vehicle; MA, obese mice + astaxanthin; FBG, fasting blood glucose; LPS, lipopolysaccharide. Astaxanthin was dissolved in corn oil at a daily dose of 60 mg/kg body weight (astaxanthin equivalents) and supplemented for 30 days.*

## Discussion

In the large and complex ecosphere, the microbiome plays a great role in the flow of energy, matter, and information, which is achieved inseparably from the interaction between the microbiome (Montoya et al., 2006). Understanding the interactions among different gut microbial populations in a community and their responses to environmental changes, such as food supplements, is critical for understanding the role that food supplement plays in human health (Zhou et al., 2011). In the past few years, the development of various computing algorithms has facilitated the interpretation of people of the microbial occurrence networks. Thanks to this, more information about the interactions between the gut microbiota and the role of key microbial species in the microbial community has been learned (Liu et al., 2019). In this study, the functional microbial co-occurrence networks were identified with the high-throughput 16S rRNA sequencing of fecal samples from the colon of mice with the supplement of astaxanthin under both normal and obese conditions.

Exploring the influence of food supplements on gut microbial composition, co-occurrence patterns, and microbial correlations with physiological parameters in a larger sample range makes the role of the food supplement in human health more credible. In this study, the gut microbiota from normal and obese mice were taken as a whole, and the effects of astaxanthin on microbial communities were studied. On this basis, the effect of astaxanthin on gut microbiota in normal and obese mice was explored separately. The intake of astaxanthin altered the composition and microbial co-occurrence patterns, and compared with normal body, the changes in microbiota were more profound under the obese condition. At the phylum level, the Firmicutes/Bacteroidetes ratio, a possible hallmark for obesity (Zou et al., 2020), was significantly decreased after the intake of astaxanthin in obese mice. At the genus level, the abundance of *Acinetobacter*, a species which represents opportunistic bacteria (Wong et al., 2017), was decreased, and the abundance of *Alistipes*, which was reported to be decreased under obese condition (Thingholm et al., 2019), was significantly increased in group A. The same changes of the abundance of the two genera occurred in the MA group compared to MO. Under normal condition, the treatment of astaxanthin decreased the abundance of *Listeria*, a kind of pathogen causing listeriosis, a severe disease with high hospitalization and case fatality rates (Maudet et al., 2021). At the OTU level, the supplement of astaxanthin significantly affected the abundance of OTUs from the order Clostridiales—for example, OTU #780650, assigned to Clostridiaceae, which was significantly decreased in group A compared to group O, was also significantly decreased by astaxanthin under healthy and obese conditions, respectively. The higher percentage of Clostridiales was associated with increasing insulin resistance (Chambers et al., 2019), intestinal inflammation (Kolho et al., 2015), and poorer cognitive flexibility (Magnusson et al., 2015). In addition, an OTU (GreenGeneID #325850) from the order RF32, which was significantly increased in its abundance by astaxanthin in group A, was also significantly increased under obese condition. The increased abundance of RF32 was related to the reduced blood high-density lipoprotein cholesterol and low-density lipoprotein cholesterol levels (*p* < 0.05) and restored liver steatosis (Rong et al., 2016).

The PICRUSt algorithm provides convenience for us to infer the functional categories of gut microbiota affected by astaxanthin. The abundance of genes in the pathways related to carbohydrate metabolism (polyketide sugar unit biosynthesis, butanoate metabolism, and propanoate metabolism), LPS biosynthesis, and intestinal integrity (membrane and intracellular structural molecules) was significantly affected by astaxanthin in obese mice. Although the prediction results have been verified by simply detecting the LPS and FBG levels, more accurate and more detailed biological function of the fecal microbial community in response to astaxanthin can be estimated by increasing the gut microbial sequencing depth and combining more predictive tools.

Topologically, different OTUs play distinct roles in the network (Guimerà et al., 2007). In this study, 96.6% of the nodes in the global networks were peripherals, and 1.9% of the OTUs were module hubs. An OTU (GreenGeneID# 589071) belonging to *Bacteroides uniformis* was identified as a module hub in group MO. It has been reported that *B. uniformis* was positively correlated with LPS levels and pathophysiological features (Xie et al., 2016). Moreover, a significant reduction of *B. uniformis* was observed in group MA. The gut microbiota contained in the family S24-7 plays a dominant role in the mouse gut microbiota, and they have also been found in the intestine of other animals (Lagkouvardos et al., 2019). An OTU (GreenGeneID# 269902) assigned to the family S24-7 was observed to serve as a module hub in both the NO and MO groups. Of note is that an OTU (GreenGeneID# 275218) from Ruminococcaceae behaved like module hubs in both the MA and A groups. Ruminococcaceae, a kind of commensal bacterial microbiota, was reported to have an effect of relieving HFD-induced obesity (Zhao et al., 2017). The loss of it has also been found in acute and chronic intestinal diseases, and a correlation with metabolic changes—for example, the bacterial metabolites related to immune regulation—was reported previously (Suchodolski, 2016). A few nodes, approximately 1.5%, acted as connectors in the global networks. These nodes are highly linked to several modules. An OTU (GreenGeneID# 322062) from the family Lachnospiraceae was identified as a connector species in group NO. Lachnospiraceae plays a critical role in the microbial community. It has been colonized in the intestinal lumen from birth, and the richness and abundance have been constantly increasing in the life of the host (Vacca et al., 2020). The supplement of astaxanthin significantly increased the abundance of Lachnospiraceae in the NA group. In addition, an OTU (GreenGeneID# 4029632) assigned to the genus *Bacteroides*, which plays a generally beneficial role in the gut (Rios-Covian et al., 2017), was observed to serve as a connector in both the MA and A groups.

The correlation between individual microbial taxa and physiological parameters in response to astaxanthin was reported in β-carotene oxygenase 2 knockout or alcoholic fatty liver mice (Liu et al., 2018, 2020). In this study, the module-based microbiota was studied to correlate the physiological parameters (FBG and LPS) with individual modules after the supplement of astaxanthin, and the functional microbial co-occurrence networks were identified. Module O02 displayed a significant positive correlation with both FBG and LPS values. Among the 62 members consisting of module O02, 47 of them were assigned to the order Clostridiales. In addition, under obese condition, module MO09 showed a significant positive correlation with LPS level. Module MO09 contained a total of 10 members, of which at least 3 OTUs assigned to Clostridiales were significantly increased in obese bodies compared to the NO group. In group MO, a positive correlation between the family Clostridiaceae and LPS value was assessed by Mantel test. It has been reported in various studies that FBG and LPS play important roles in glucose intolerance, inflammation, and other diseases. The analysis results mentioned above indicated the role of Clostridiales as a potential pathological marker. Well-designed experiments are needed to verify the microbial interactions inferred by network tools, which may reveal the mechanisms of action with astaxanthin supplements. It can be seen from the study that the topological roles, modularity, module memberships, and interaction patterns are rich sources of new hypotheses for understanding the interactions among different gut microbial populations and identifying key microbial populations in response to environmental changes, such as food supplements, in human microbial communities.

## Data Availability Statement

The datasets presented in this study can be found in online repositories. The names of the repository/repositories and accession number(s) can be found below: NCBI SRA BioProject, accession no: PRJNA729231.

## Ethics Statement

The animal study was reviewed and approved by the Animal Ethics Committee of the Ocean University of China.

## Author Contributions

YG and QT designed the experiments. YG, FL, and RL performed the experiments. YG, FL, and CL analyzed and interpreted the data. YG wrote the manuscript. QT revised the manuscript. QT and CX supervised the experiments. All the authors checked the manuscript and the submitted final version.

## Conflict of Interest

The authors declare that the research was conducted in the absence of any commercial or financial relationships that could be construed as a potential conflict of interest.

## Publisher’s Note

All claims expressed in this article are solely those of the authors and do not necessarily represent those of their affiliated organizations, or those of the publisher, the editors and the reviewers. Any product that may be evaluated in this article, or claim that may be made by its manufacturer, is not guaranteed or endorsed by the publisher.

## Footnotes

1
http://ieg4.rccc.ou.edu/mena/
