# Supplementary material for: Microbial Composition and Co-occurrence Patterns in the Gut Microbial Community of Normal and Obese Mice in Response to Astaxanthin
Source: Front Microbiol. 2021 Sep 6;12:671271. doi: 10.3389/fmicb.2021.671271 (PMC8450573; doi:10.3389/fmicb.2021.671271)
Supplement: Supplementary file 1 [file Data_Sheet_1.ZIP › supplementary materials/Table S1.docx]

**Table S1.** Composition of Rodent Diets

| **diet** | **NC** | | **HFD** | |
| --- | --- | --- | --- | --- |
| *product #* | *D12450J* | | *D12451* | |
|  | *gm*% | *kcal*% | *gm*% | *kcal*% |
| protein | 19.2 | 20 | 24 | 20 |
| carbohydrate | 67.3 | 70 | 41 | 35 |
| fat | 4.3 | 10 | 24 | 45 |
| total |  | 100 |  | 100 |
| kcal/gm | 3.85 |  | 4.73 |  |
| *ingredient* | *gm* | *kcal* | *gm* | *kcal* |
| casein | 200 | 800 | 200 | 800 |
| L-cysteine | 3 | 12 | 3 | 12 |
| corn starch | 506.2 | 2024.8 | 72.8 | 291 |
| maltodextrin 10 | 125 | 500 | 100 | 400 |
| sucrose | 68.8 | 275.2 | 172.8 | 691 |
| cellulose, BW200 | 50 | 0 | 50 | 0 |
| soybean oil | 25 | 225 | 25 | 225 |
| lard | 20 | 180 | 177.5 | 1598 |
| mineral mix, S10026 | 10 | 0 | 10 | 0 |
| dicalcium phosphate | 13 | 0 | 13 | 0 |
| calcium carbonate | 5.5 | 0 | 5.5 | 0 |
| potassium citrate, 1 H_2_O | 16.5 | 0 | 16.5 | 0 |
| vitamin mix, V10001 | 10 | 40 | 10 | 40 |
| choline bitartrate | 2 | 0 | 2 | 0 |
| **total** | **1055** | **4057** | **858.1** | **4057** |

NC, normal control diet; HFD, high-fat and high-sucrose diet.
