# Supplementary material for: Microbial Composition and Co-occurrence Patterns in the Gut Microbial Community of Normal and Obese Mice in Response to Astaxanthin
Source: Front Microbiol. 2021 Sep 6;12:671271. doi: 10.3389/fmicb.2021.671271 (PMC8450573; doi:10.3389/fmicb.2021.671271)
Supplement: Supplementary file 1 [file Data_Sheet_1.ZIP › supplementary materials/Table S3.docx]

**Table S3.** Topological properties of the global network inferred using a Random-Matrix theory based network pipeline under various experimental conditions.

| **Network Indexes** | **O** | **A** | **NO** | **NA** | **MO** | **MA** |
| --- | --- | --- | --- | --- | --- | --- |
| Total nodes | 381 | 325 | 447 | 420 | 566 | 393 |
| Total links | 581 | 411 | 751 | 493 | 866 | 587 |
| Total modules | 39 | 37 | 38 | 50 | 62 | 43 |
| Modularity | 0.731 | 0.773 | 0.827 | 0.885 | 0.864 | 0.790 |
| R square of power-law | 0.936 | 0.922 | 0.891 | 0.859 | 0.913 | 0.853 |
| Average degree (avgK) | 3.05 | 2.529 | 3.36 | 2.348 | 3.06 | 2.987 |
| Average clustering coefficient (avgCC) | 0.112 | 0.088 | 0.183 | 0.138 | 0.163 | 0.141 |
| Average path distance (GD) | 5.781 | 6.222 | 8.018 | 9.798 | 8.43 | 6.605 |
| Geodesic efficiency (E) | 0.209 | 0.194 | 0.159 | 0.133 | 0.152 | 0.186 |
| Harmonic geodesic distance (HD) | 4.774 | 5.166 | 6.275 | 7.501 | 6.562 | 5.385 |
| Maximal degree | 20 | 12 | 21 | 15 | 27 | 20 |
| Nodes with max degree (GreenGeneID) | 189489 | 761408 | 1110135 | 198753 | 269902 | 275218 |
| Centralization of degree (CD) | 0.045 | 0.029 | 0.04 | 0.03 | 0.043 | 0.044 |
| Maximal betweenness | 8439.552 | 6129.415 | 21583.36 | 22583.01 | 25212.74 | 14974.85 |
| Nodes with max betweenness (GreenGeneID) | 189489 | 269949 | 839598 | 198753 | 269902 | 275218 |
| Centralization of betweenness (CB) | 0.109 | 0.107 | 0.206 | 0.246 | 0.151 | 0.186 |
| Maximal stress centrality | 59277 | 20225 | 156639 | 78189 | 558453 | 76392 |
| Nodes with max stress centrality (GreenGeneID) | 189489 | 323614 | 839598 | 198753 | 170926 | 275218 |
| Centralization of stress centrality (CS) | 0.776 | 0.357 | 1.474 | 0.856 | 3.378 | 0.951 |
| Maximal eigenvector centrality | 0.341 | 0.374 | 0.351 | 0.517 | 0.323 | 0.327 |
| Nodes with max eigenvector centrality (GreenGeneID) | 189489 | 414089 | 1110135 | 198753 | 268733 | 378273 |
| Centralization of eigenvector centrality (CE) | 0.322 | 0.35 | 0.34 | 0.506 | 0.312 | 0.31 |
| Density (D) | 0.008 | 0.008 | 0.008 | 0.006 | 0.005 | 0.008 |
| Reciprocity | 1 | 1 | 1 | 1 | 1 | 1 |
| Transitivity (Trans) | 0.125 | 0.105 | 0.233 | 0.181 | 0.173 | 0.216 |
| Connectedness (Con) | 0.634 | 0.631 | 0.75 | 0.592 | 0.581 | 0.66 |
| Efficiency | 0.991 | 0.992 | 0.993 | 0.994 | 0.993 | 0.992 |
| Hierarchy | 0 | 0 | 0 | 0 | 0 | 0 |
| Lubness | 1 | 1 | 1 | 1 | 1 | 1 |

O: normal or obese mice + corn oil vehicle; A: normal or obese mice + astaxanthin; NO: normal mice + corn oil vehicle; NA: normal mice + astaxanthin; MO: obese mice + corn oil vehicle; MA: obese mice + astaxanthin. Astaxanthin was dissolved in corn oil at a daily dose of 60 mg/kg body weight (astaxanthin equivalents) and supplemented for 30 days.
